# Patterns of Plant Biomass Partitioning Depend on Nitrogen Source

**DOI:** 10.1371/journal.pone.0019211

**Published:** 2011-04-22

**Authors:** Camila Aguetoni Cambui, Henrik Svennerstam, Linda Gruffman, Annika Nordin, Ulrika Ganeteg, Torgny Näsholm

**Affiliations:** 1 Department of Forest Genetics and Plant Physiology Swedish University of Agricultural Sciences, Umeå, Sweden; 2 Department of Forest Ecology and Management, Swedish University of Agricultural Sciences, Umeå, Sweden; University of Leipzig, Germany

## Abstract

Nitrogen (N) availability is a strong determinant of plant biomass partitioning, but the role of different N sources in this process is unknown. Plants inhabiting low productivity ecosystems typically partition a large share of total biomass to belowground structures. In these systems, organic N may often dominate plant available N. With increasing productivity, plant biomass partitioning shifts to aboveground structures, along with a shift in available N to inorganic forms of N. We tested the hypothesis that the form of N taken up by plants is an important determinant of plant biomass partitioning by cultivating *Arabidopsis thaliana* on different N source mixtures. Plants grown on different N mixtures were similar in size, but those supplied with organic N displayed a significantly greater root fraction. ^15^N labelling suggested that, in this case, a larger share of absorbed organic N was retained in roots and split-root experiments suggested this may depend on a direct incorporation of absorbed amino acid N into roots. These results suggest the form of N acquired affects plant biomass partitioning and adds new information on the interaction between N and biomass partitioning in plants.

## Introduction

Plants adjust to variations in resource availabilities by variable partitioning to root and shoot growth [1], [2]. Among various edaphic factors that affect plant biomass partitioning, nitrogen (N) is particularly important; higher rates of available N shift partitioning from roots to shoots [3]–[7]. Studies of partitioning responses to shifts in N supply have revealed a positive linear relationship between shoot∶root ratios and the internal N status of plants [8]–[10]. Plants growing on infertile, low N soils are also reported to have a higher root mass fraction than plants growing on more fertile and N rich soils [11]–[12]. Thus, in natural ecosystems, soil N availability and plant biomass partitioning exhibit strong co-variation.

However, there is quantitative and qualitative variation in soil N availability in most ecosystems [13]. In poor soils of natural ecosystems of the arctic, boreal [14]–[18] and alpine [19], [20] regions, organic N predominates. In contrast, an increasing share of mineral N, particularly NO_3_^−^, is found in soils of temperate [21] and subtropical [22]–[23] regions, particularly in agricultural soils [24], [25]. The well-documented effect of soil N availability on regulating plant biomass partitioning may thus, in natural ecosystems, be confounded by changes in the forms of N in soil solution.

Optimisation of biomass partitioning is thought to minimise the stress imposed by the limiting resource, in this case N. Arguably, such optima must depend on the actual chemical form(s) of N present for uptake, because plant N acquisition is mainly limited by processes through which N sources come into contact with root surfaces: mass flow induced by transpiration, diffusion and interception [26]. Nitrate can be efficiently acquired through mass flow because of its high mobility in soils. Organic N compounds are less mobile in soil solution [25] and hence may mainly be acquired through diffusion. This implies that, for similar rates of N uptake, plants would need to partition a larger share of biomass to roots (or mycorrhizal hyphae) when N is available as organic N, compared with conditions when NO_3_^−^ is the dominant N source. Thus, for a given total N availability, an optimum for biomass partitioning in a NO_3_^−^-dominated environment should be different and with a higher shoot∶root ratio, compared with the corresponding optimum with organic N as the dominant N source.

In a study of *Pinus sylvestris* seedlings [27], it was shown that the short-term distribution of N varied for different N sources. Thus, in this study, N absorbed as NO_3_^−^ was to a higher extent allocated to shoots compared to N absorbed as organic forms. Whether such short-term differences in N allocation between different N forms are relevant for the long-term distribution of N is, however, not known. As discussed above, plants in natural environments usually encounter a range of organic and inorganic N compounds simultaneously and may thus absorb and utilize several different forms of N. Under such conditions, mechanisms for tuning biomass partitioning to both soil N availability and soil N chemical composition would be of significant value. One possible mechanism would be if the different chemical forms of absorbed N were distributed within the plant in line with the optimal partitioning of biomass for that particular form of N. Thus, N absorbed as NO_3_^−^ would be partitioned to a larger extent to shoots while N absorbed in organic forms to a larger extent to roots and this would offer a mechanism through which plants could optimise biomass partitioning according to the chemical composition of soil N.

We hypothesised that the chemical forms of N that plants take up would affect biomass partitioning between shoots and roots. Furthermore, we hypothesised that the internal distribution of absorbed N would depend on which form of N is absorbed. So, for example, absorbed organic N would be retained in roots to a greater extent than absorbed inorganic N. We used a sterile growth system with agar as a root medium and cultivated *Arabidopsis thaliana* with mixtures of different N sources, in order to study how N forms affected plant biomass partitioning. We traced the origin of total plant N, as well as the origin of root and shoot N, from individual N sources using ^15^N-labelling. We also investigated the movement of different N sources between plant parts.

## Materials and Methods

### Experiment 1. Growth and biomass partitioning on different N source mixtures

Wild type (ecotype Col-0) *Arabidopsis thaliana* (Arabidopsis) were grown on sterile agar plates containing the equivalent of N-free half-strength Murashige and Skoog (MS) medium [28], with 0.65% w/v agar (plant agar, Duchefa Biochemie), 0.5% w/v sucrose and the pH set to 5.8 with MES buffer. Nitrogen was administered as one of the following N source mixtures: (1) 6 mM NO_3_^−^; (2) 3 mM NH_4_NO_3_; (3) 1 mM glutamine+4 mM NO_3_^−^; (4) 1.5 mM glutamine+3 mM NO_3_^−^ and (5) 2 mM glutamine+2 mM NO_3_^−^. Thus, all treatments included 6 mM N and mixtures of glutamine and NO_3_- were in the ratios 1∶2; 1∶1 and 2∶1. Sterile filtered glutamine was added to the agar mixture after autoclaving, to ensure that it was intact in the medium. Plates were filled with 40 ml of agar and five seeds were sown onto each plate. Agar plates were incubated in a cold room for two days to synchronize germination and then transferred to a growth cabinet with a 16/8 h light/dark (200 µmoles photons m^−2^ s^−1^) and 23/18°C (day/night) regime. Plant shoots were not in contact with the agar surface, so all N in plants was derived from root uptake. Plants were harvested after 21 days. At harvest, roots and shoots were dried at 60°C for 24 hours and weighed.

### Experiment 2. Growth, biomass partitioning and ^15^N labelling

Arabidopsis plants were cultivated as in experiment 1 but in this experiment, N was administered either as 3 mM NH_4_NO_3_ or as 1.5 mM gln+3 mM NO_3_^−^ in the media. Thus, both mixtures had a total N concentration of 6 mM. In each growth unit, one of the N sources was ^15^N labelled and for each N source mixture, reciprocal labelling was performed. Thus, for the NH_4_NO_3_ mixture, five plates were labelled with 1.0 atom % ^15^NH_4_^+^ and five with 1.0 atom % ^15^NO_3_^−^. Similarly, for the glutamine+NO_3_^−^ mixture, five plates were labelled with 1.0 atom % α-^15^N-glutamine and five with 1.0 atom % ^15^NO_3_^−^. Plants were harvested after 21 days. Roots were rinsed and cleaned thoroughly three times in 0.5 mM CaCl_2_ to remove ^15^N labelled compounds from surfaces. Roots and shoots were dried at 60°C for 24 hours, weighed and homogenised. Finally, samples were analysed using a Europa Scientific Isotope Ratio Mass Spectrometer to determine total N and ^15^N contents. For each labelled N source, five replicate plates were used (n = 5).

In a separate experiment, Arabidopsis plants were grown as described above, but with N supplied as 3 mM NO_3_^−^+30 µM U^15^N_4_, U^13^C_6_-L-Arg (>98% ^15^N; corresponding to 120 µM L-Arg-N). The ^15^N-labelling enabled analysis of the distribution of absorbed arginine-N. ^13^C labelling was included to allow analysis of distribution of absorbed arginine-C but this part of the study was later discontinued since it was realized that the risk for re-fixation of respired arginine-^13^C through photosynthesis would lead to non-conclusive results. Harvests and analysis of total N and ^15^N content of shoots and roots were performed as described above. Five plants were grown on each plate and four plates were harvested (n = 4) for each measurement.

### Experiment 3. Split root experiment

*Arabidopsis thaliana* (ecotype Col-0) seeds were surface-sterilized, exposed to 4°C during 48 h (to synchronise germination) and cultivated for 11–14 days on vertical plates containing half-strength N-free Murashige and Skoog (MS) medium [28], with 3 mM KNO_3_, 0.5% w/v sucrose, 1% w/v agar (plant agar, Duchefa Biochemie), buffered to pH 5.8 with 7.7 mM MES. Plants were grown in climate chamber under short-day conditions (light/dark period of 8/16 hours), temperature regime of 22/18°C degrees and light intensity of 200 µmoles photons m^−2^ s^−1^ in order to avoid early flowering. Flowering causes internal re-distribution of N, which would complicate any assessment of the internal N fluxes absorbed as different sources. After 2 weeks of growth, the primary roots of plants were removed to stimulate lateral root development and, one week afterwards, when the new roots were approximately 2–4 cm long, the plants were transferred to a split-root experiment system. This system consisted of Petri dishes with two separate root-growth compartments.

Two trials were conducted with this setup. In the first, both cells of the plate contained the equivalent of N-free half strength MS medium and N was administered as a mixture of 3 mM NO_3_^−^ +1.5 mM glutamine. In one of the halves, one of the N sources (either NO_3_^−^ or glutamine) was ^15^N labelled at a rate of 1.0 atom % excess. Transferred plants (n = 6 for plants grown on mixtures where the glutamine was labelled and n = 7 for plants grown on mixtures where the NO_3_^−^ was labelled) were placed on the mid rib and their root systems divided into two similar fractions that were positioned on either side of the rib. After two weeks of growth in this system, plants were harvested and shoot and roots from the two compartments were separated.

 In the second trial, the two compartments were filled with half-strength N-free MS medium and N was supplied as 3 mM NO_3_^−^ on one of the root compartments while on the other half, N was supplied as 1.5 mM glutamine. Potassium was compensated in the glutamine treatment with the equivalent addition of KCl. For each plate, one of the N sources was labelled with 1 atom % ^15^N excess (five plates containing 1% ^15^NO_3_^−^ and five plates with 1 atom % excess ^15^N-glutamine). Plants were grown for 14 days before harvest.

At harvest, all samples were washed 3 times with 0.5 mM CaCl_2_ to remove N compounds adhering to root surfaces, dried at 60°C and homogenized for determination of total N and ^15^N content. Analyses were conducted using a Europa Scientific Isotope Ratio Mass Spectrometer.

### Statistics

Significant differences between N treatments and between plant parts were tested using ANOVA followed by Tukey's post hoc test.

## Results

**Figure 1:**
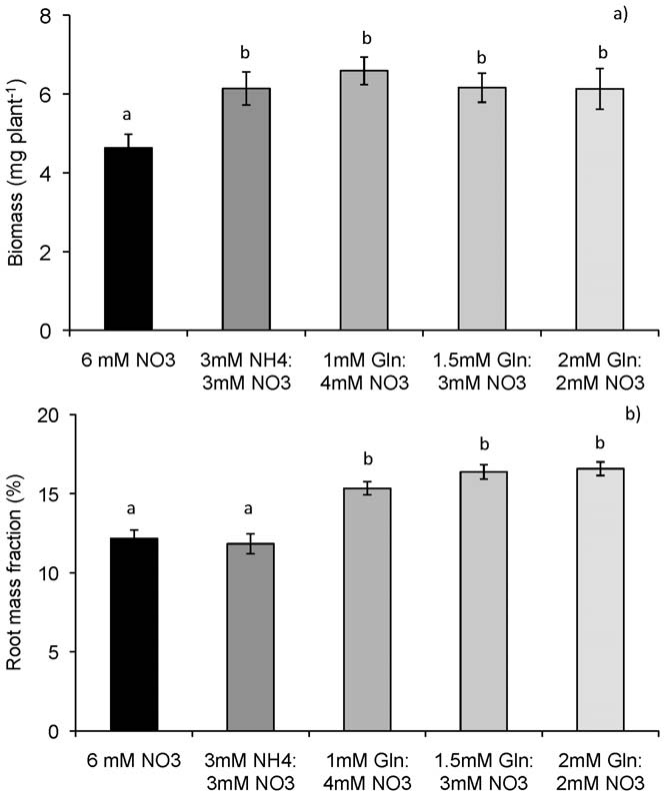
Biomass (a) and fraction of biomass in roots (b) of *Arabidopsis thaliana* grown on either NO_3_^−^ or on NH_4_NO_3_ or on different combinations of glutamine and NO_3_^−^. All media had a total N concentration of 6 mM. Plants were grown on sterile agar plates for 21 days. Bars represent average values ± SE, n = 8. Different lower-case letters indicate differences at p≤0.05 between N treatments.

In the first experiment, we tested how various mixtures of glutamine and NO_3_^−^ affected growth and biomass partitioning in Arabidopsis, as compared with pure NO_3_^−^ and NH_4_NO_3_. The rationale behind this experiment was to test whether an increasing fraction of organic N in the growth media would correspond to an increased fraction of plant biomass partitioned to roots. Plants grown on NO_3_^−^ were significantly smaller than the other N treatments. A significant effect of N source on the root mass fraction was found; the root fractions of plants grown on any of the glutamine mixtures were in all cases significantly higher than those of plants grown on NH_4_NO_3_ or NO_3_^−^ (Fig. 1). The root mass fraction did, however, not display a significant increase with an increasing share of glutamine in the growth media.

In the second experiment, we used ^15^N-labelled N sources to assess plant uptake and internal distribution of individual N compounds in the mixtures. This enabled us to test if an effect of a specific N source on biomass partitioning was paralleled by a specific pattern of distribution of N from that source within the plant.

**Figure 2:**
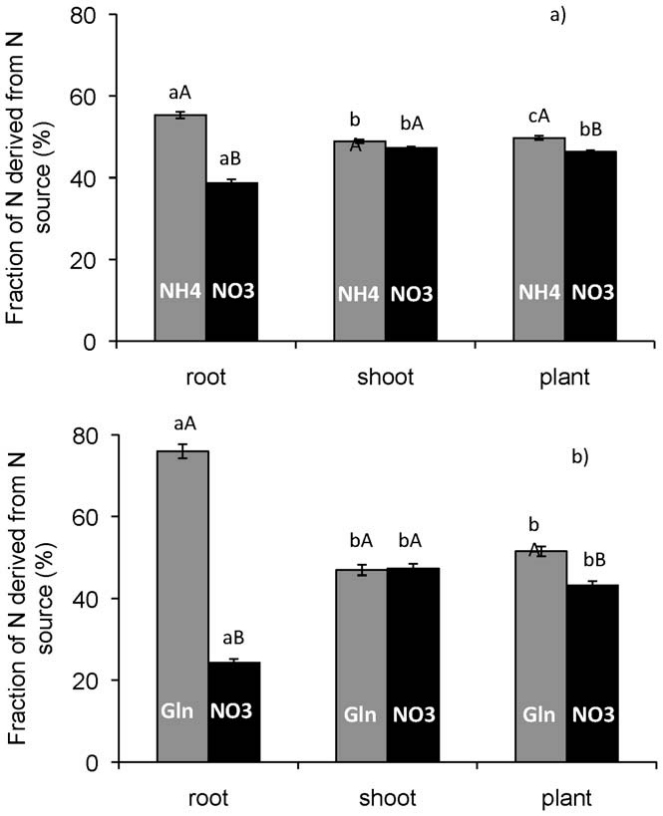
Origin of root N, shoot N and plant N, in *Arabidopsis thaliana* plants grown on 3 mM NH_4_NO_3_ (a) or a mixture of 1.5 mM glutamine+3 mM NO_3_^−^ (b). Fractions of N derived from individual N sources in the mixtures were calculated from N contents and rates of ^15^N abundance in plant parts. Plants were grown on sterile agar plates for 21 days. Bars represent average values ± SE, n = 5. Different lower-case and capital letters indicate differences at p≤0.05 between plant parts and between N sources, respectively.

Plants supplied with mixtures of glutamine and NO_3_^−^ (^15^Ngln-NO_3_ and gln-^15^NO_3_) had higher biomass, root biomass and a higher root mass fraction than plants supplied with NH_4_NO_3_ (Table 1). No statistical difference in N concentration of plants from the different treatments was found. The abundance of ^15^N in plant parts was clearly different for plants grown on glutamine and NO_3_^−^ mixtures, but this difference was smaller for plants grown on NH_4_NO_3_ (Table 2). δ^15^N of shoots of plants supplied ^15^Ngln-NO_3_ was lower than any of the other three treatments (gln-^15^NO_3_, ^15^NH_4_NO_3_ and NH_4_^15^NO_3_). These differences suggested that root N was derived more from uptake of glutamine than from NO_3_^−^, while the opposite was true for shoot N. Values of ^15^N abundances were therefore used to calculate fractions of plant, root and shoot N that were derived from each individual N source (Fig. 2). Arabidopsis plants grown on NH_4_NO_3_ had 50% N content derived from NH_4_^+^ and 46% derived from NO_3_^−^. Thus, 96% of N in the biomass was accounted for by uptake of the two N sources in these plants. For root N, 55% was derived from NH_4_^+^ and 39% from NO_3_^−^. For shoot N, 49% was derived from NH_4_^+^ and 47% from NO_3_^−^, i.e. compared with total plant N, N derived from NO_3_^−^ was slightly more abundant in shoots (Fig. 2a). The ratio between N derived from NH_4_^+^ and that derived from NO_3_^−^ was thus 1.4, 1.0 and 1.1 for, root N, shoot N and total plant N, respectively. The biomass of roots were always much smaller than that of shoots (cf. Table 1) and hence a high abundance of N derived from organic N in roots still had a limited effect on the fraction of total plant N derived from organic N.

**Table 1:** Biomass (mg plant^−1^), N concentrations (mg g^−1^) and root mass fraction of plants grown in sterile agar culture and with N supplied as either a mixture of 1.5 mM glutamine and 3 mM NO_3_^−^ or 3 mM NH_4_NO_3_.

N-source	Root biomass	Root N conc	Shoot biomass	Shoot N conc	Total biomass	Root mass fraction
Gln-NO_3_	1.2±0.1 (a)	49±2 (a)	5.1±0.3 (a)	57±2 (a)	6.3±0.3 (a)	0.19±0.0 (a)
NH_4_NO_3_	0.8±0.05 (b)	48±1 (a)	4.7±0.1 (a)	59±1 (a)	5.5±0.1 (b)	0.14±0.0 (b)

Average values ± SE, n = 5. Different letters indicate differences at p≤0.05 between N treatments.

**Table 2:** δ^15^N of Arabidopsis roots and shoots of plants grown in sterile agar culture and with N supplied as either a mixture of 1.5 mM glutamine and 3 mM NO_3_^−^ or 3 mM NH_4_NO_3_.

N-treatment	Root δ ^15^N	Shoot δ^15^N
^15^Gln-NO_3_	1034±53 (a, A)	639±40 (a, B)
Gln-^15^NO_3_	657±53 (b, A)	1297±32 (b, B)
^15^NH_4_NO_3_	1526±54 (c, A)	1351±28 (b, B)
NH_4_^15^NO_3_	1066±56 (a, A)	1305±21 (b, B)

In these mixtures, one of the added N sources was labelled with ^15^N at a rate of 1 atom %. Average values ± SE, n = 5. Different lower-case and capital letters indicate differences at p≤0.05 between N treatments, and between plant parts, respectively.

The N content of Arabidopsis plants grown on mixtures of NO_3_^−^ and glutamine also reflected the different N sources. N derived from glutamine accounted for 52% and N derived from NO_3_^−^ accounted for 43% of plant N. Thus, in these plants, 95% of total plant N was accounted for by uptake of the two N sources. Nitrogen not accounted for in these measurements would to some extent result from the N contained in seeds but could also result from a somewhat lower ^15^N abundance of tracers compared to those given by the manufacturer. Small variations in the ^15^N abundance of the non-labelled compounds could also be part of the explanation of the N not accounted for in the ^15^N mass-balance calculations. Significantly more root N was derived from glutamine (76%) and significantly less from NO_3_^−^ (24%), compared with total plant N. Shoot N was derived equally much from glutamine (47%) and from NO_3_^−^ (47%), but compared with total plant N, N derived from glutamine was less represented in shoots (Fig. 2b). The ratio between N derived from glutamine and N derived from NO_3_^−^ was thus 3.1, 1.0 and 1.2 for root N, shoot N and total plant N, respectively.

We then asked if the observed preferential distribution of N derived from uptake of glutamine was specific for this particular N source or if N derived from other amino acids would show similar patterns of distribution between shoot and roots. Thus, in an additional experiment, we grew plants as described above but with N supplied as 3 mM NO_3_^−^+30 µM of labelled L-arginine. This mixture enabled tracing of absorbed arginine-N but avoided the problem of growth inhibition of L-arginine that occurs at higher rates [29]. ^15^N-values of roots and shoots from plants grown on the 3 mM NO_3_^−^+30 µM arginine mixture were (average ± SE) 5.2±0.08 and 2.7±0.08 atom % respectively. Thus, N derived from L-arginine uptake was more than twice as abundant in roots as in shoots, suggesting N derived from uptake of arginine displays a similar pattern of preferential allocation to roots as does glutamine.

In the third experiment, we aimed at studying the movement of absorbed glutamine-N and NO_3_^−^-N between plant parts. We therefore established a split-root system and used this for two different trials. In the first trial, roots on both sides of a rib were supplied with identical mixtures of glutamine and NO_3_^−^, but one of these N sources was ^15^N-labelled on just one side of the rib. This experiment tested if the overrepresentation of glutamine-N in roots (Fig. 2b) was primarily a result of it being incorporated at the site of uptake or if absorbed glutamine-N was primarily allocated to growth of roots, irrespective of the site of absorption. Overrepresentation is here defined as when the ratio fraction of N derived from one N source of a specific tissue : fraction of N derived from that N source of the plant >1.

**Figure 3 pone-0019211-g003:**
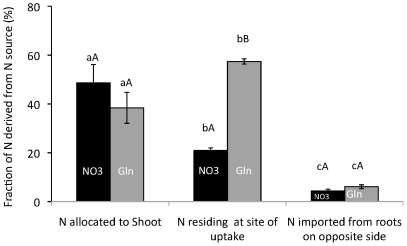
Split-root experiment with *Arabidopsis thaliana*. Plants were grown on agar plates that were divided into two identical compartments by a plastic rib. The growth medium was identical on both sides of the rib and with N supplied as a mixture of 1.5 mM glutamine+3 mM NO_3_^−^ but on one side, one of the N sources (either glutamine or NO_3_^−^) was ^15^N-labelled. Bars indicate the fraction of N derived from each source and represent average ± SE, n = 6–7. Different lower-case and capital letters indicate differences at p≤0.05 between plants parts and between N sources, respectively.

The fraction of shoot-N derived from uptake of NO_3_^−^ (49%) was higher than that of labelled glutamine (38%; Fig. 3). The fraction of root N residing at the site of uptake was 57% and 21% for glutamine and NO_3_^−^ respectively while the fraction of root N imported from the opposite side of the plate was low for both N sources: only 6% and 4% (Fig. 3).

**Figure 4:**
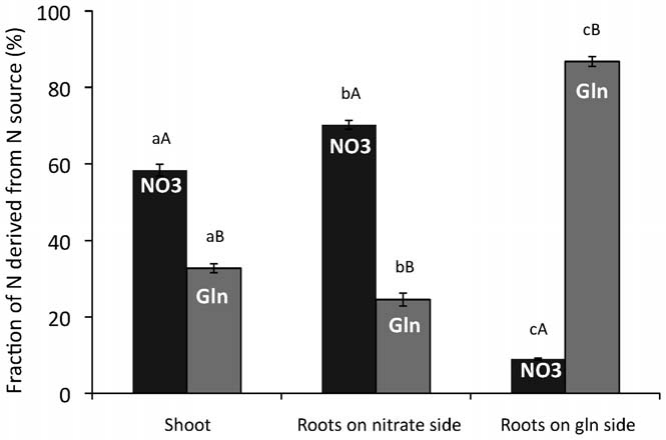
Split-root experiment with *Arabidopsis thaliana*. Plants were grown on agar plates that were divided into two identical compartments by a plastic rib. The two compartments contained either 1.5 mM glutamine or 3 mM NO_3_^−^ as N sources. For each plate, one of the N sources (either glutamine or NO_3_^−^) was ^15^N-labelled. Bars indicate the fraction of N derived from each source for the shoot and for roots growing in the NO_3_^−^ compartment and the glutamine compartment. Bars represent average ± SE, n = 5. Different lower-case and capital letters indicate differences at p≤0.05 between plant parts, and between N sources, respectively.

In the second split-root trial, the two root compartments contained different N sources; NO_3_^−^ on one side and glutamine on the other. This enabled assessment of how different N sources are allocated between the two parts of the root system and to the shoot. A clear difference was observed in the partitioning of N from NO_3_^−^ and from glutamine by the plant (Fig. 4). Thus, shoot N was to 58% and 33% derived from NO_3_^−^ and glutamine respectively while root N on the glutamine side was to 87% derived from glutamine and 9% from NO_3_^−^. Root N on the NO_3_^−^ side was to 70% derived from NO_3_^−^ and to 25% from glutamine. Thus, N derived from glutamine was less abundant in shoots but more abundant in roots and also showed a greater tendency for translocation to parts of the root system that grew in a NO_3_^−^ medium Fig. 4).

## Discussion

Numerous studies have shown how soil N availability affects plant biomass partitioning [3]–[5], [8]–[10]. These reports have, however, largely neglected any potential role of the chemical species of available N in the soil but see [6]. We hypothesised that plant biomass partitioning is linked to the actual source(s) of N available for uptake. The rationale underpinning this hypothesis is that plants growing on soils where N is mainly present in the form of ammonium and/or organic forms should at similar rates of N availabilities, theoretically require a larger root (or mycorrhizal) surface area for N acquisition compared to when a fraction of soil N is present in the form of NO_3_^−^ simply because of the difference in mobility displayed by different N sources [24]. Our results show that inclusion of an amino acid in the growth medium resulted in higher (Table 1) total biomass accumulation and that biomass partitioning to roots in all cases was significantly enhanced (Fig. 1). This result is clearly in contradiction to the general idea that plants at similar rates of N supply should exhibit similar patterns of biomass partitioning [8]. The interdependency of internal N status, relative growth rate and biomass partitioning has been reported [9]. Thus, comparisons of biomass partitioning are only valid for plants displaying similar growth rates and internal N status. In the current study, plant N was not significantly different for plants grown on different N source mixtures (Table 1) and thus, we are able to compare how individual N forms affect biomass partitioning of plants. Earlier studies of the effects of different N forms on biomass partitioning have mostly compared NH_4_^+^ and NO_3_^−^ [6] and references therein. However, root growth is often inhibited by high concentrations of NH_4_^+^ but not of NO_3_^−^ [6], which complicates comparison between these N sources. Some earlier studies have also investigated the effects of amino acids on biomass partitioning. In a study of *Catasetum fimbriatum* (*Orchidaceae*), glutamine was included as a N source [30] but in that study, plants supplied with glutamine grew nearly twice as fast as those with the other tested N sources (urea, NH_4_^+^ and NO_3_^−^), precluding direct comparison of how biomass partitioning was affected by N source. Comparisons of the effects of glutamine, NH_4_^+^ and NO_3_^−^ on biomass partitioning have also been made for *Phaseolus vulgaris*, but no significant differences were detected [31], [32]. However, in all these studies, plants were not grown in sterile culture and thus the actual contribution of glutamine to plant N uptake was unknown. In a recent article, Paungfoo-Lonhienne et al. [33] showed that Arabidopsis and *Hakea actites* can use protein as a source of N. Interestingly, they reported that root, but not shoot growth was stimulated when protein was supplied as the sole N source to plants (cf. Fig. 1 of [33].

We also hypothesized that the distribution of absorbed N between shoots and roots would differ for different N compounds. We therefore employed a stable isotope labelling approach to trace the fate of different N sources. For plants grown on mixtures of NH_4_^+^ and NO_3_^−^, a slight over-representation of N derived from NH_4_^+^ was detected in roots (Fig. 2a). However, for plants grown on mixtures of glutamine and NO_3_^−^, we found a significant over-representation of N derived from the organic source in roots: as much as 76% of root N was derived from absorbed organic N (Fig. 2b). Thus, the increased root mass fraction of plants supplied with glutamine in the growth media was paralleled by a large share of root N derived from the uptake of glutamine. Results of tests with Arabidopsis grown on 3 mM NO_3_^−^ and supplied with 30 µM ^15^N (96–98 atom %) labelled arginine showed over-representation of N derived from arginine in roots (5.2 and 2.7 atom % excess for roots and shoots, respectively). This supports the hypothesis that N derived from uptake of organic sources may be over-represented in roots.

The primary site of assimilation differs for NO_3_^−^ and glutamine, so that a significant share of absorbed NO_3_^−^ may be directly transported to the shoot [31], while absorbed amino acids may be preferentially metabolised in roots [27]. An over-representation of N derived from glutamine in roots may, therefore, simply reflect the difference in the site of assimilation. However, several studies have reported that absorbed N may cycle between the roots and shoots through xylem and phloem transport [34], [35]. Such N cycling is probably an important trait in plant plasticity and may, for example, enable roots to grow through patches of soil with low N availabilities [2], [36] and enable N partitioning to be controlled by developmental cues [37], [38]. Nevertheless, our results (Fig. 2–4) suggest a significant fraction of absorbed amino acid N resides, and is incorporated, at the site of primary assimilation. This would lead to the observed over-representation of N derived from glutamine in roots and the concomitant overrepresentation of NO_3_^−^-N in shoots. Over-representation of N derived from glutamine in roots may also be related to the energetic differences between the two N sources (NO_3_^−^ and glutamine). If root growth was limited by carbohydrate supply, utilisation of glutamine as a N source for the growing root would lead to appreciable energy savings [32], [37], [39]. However, Zerihun et al. [32] suggested that the importance of differences in energy requirements for utilisation of various N forms was negligible, in comparison with the costs of protein turnover. Nevertheless, this does not preclude energy savings for specific cell types or tissues, e.g. in root meristems. This is supported by studies that show a strong effect of sucrose added to growth media on root elongation rates, in particular for plants growing under low light conditions [40]. Following absorption by roots, inorganic N is assimilated into glutamine. High glutamine concentrations either resulting from high rates of synthesis or from uptake from the root medium are known to stimulate expression of the enzyme PEP-carboxylase, possibly as a means to supply 2-oxo acids, drawn from the TCA-cycle through amino acid synthesis [41]. Thus, in our experiment, PEP-carboxylase activities may be expected to be up-regulated in response to uptake of N, irrespective of in which form this N was absorbed but the input of C via uptake of glutamine would eventually counteract the depletion of oxo-acids in root cells.

The hypotheses mentioned above relate to the association between glutamine uptake and C and N use for root growth. An alternative to this model is that absorbed glutamine-N preferentially targets the growth of roots, irrespective of site of uptake. We tested this possibility in two split-root trials, the first aiming at studying patterns of N partitioning between roots growing in identical N environments, the second assessing N partitioning between roots growing on different N sources. A relatively low rate of labelling of roots in the non-labelled compartment was found for both ^15^NO_3_^−^ (4% of root N) and ^15^N-glutamine (6% of root N; Fig. 3) when roots were growing in identical N environments. This suggests that translocation of N from either N source from one side of the root system to the other is relatively small. The large accumulation of ^15^N in roots growing in the ^15^N labelled compartment (Fig. 3), compared with roots growing in the non-labelled part, suggests N absorbed by one part of the root system is, to a significant degree, also used for growth of that part of the roots irrespective of N source. However, when the two root-parts were supplied different N sources, glutamine on one side and NO_3_^−^ on the other, this pattern changed and we detected a significantly increased movement of glutamine-N over to the NO_3_^−^ side while the movement of ^15^NO_3_^−^ over to the glutamine side was small. Thus, root-N on the NO_3_^−^ side was to 25% derived from uptake of glutamine while root-N on the glutamine side was to only 9% derived from uptake of NO_3_^−^. Recalculated, a breakdown of total ^15^N label of plants supplied ^15^NO_3_^−^ showed that 84%, 15% and 1% of detected ^15^N excess was found in shoots, roots growing on the ^15^NO_3_^−^ side and roots growing on the glutamine side respectively. Corresponding figures for plants supplied ^15^N-glutamine were 72%, 20% and 8%, clearly showing the smaller contribution of glutamine-N to shoot N and the larger contribution to root N. Thus, the use of the split-root system allowed us to verify N fluxes *in planta* and the preferences of specific organs for the N sources supplied. A clear difference was observed in the use of nitrate and glutamine by plants, as both sources were not equally distributed in the organs. Thus, these two trials corroborate that N absorbed in organic form is to a larger extent used for growth of roots than of shoots compared to N absorbed as NO_3_^−^. They also show that translocation of N between different parts may be substantial from roots absorbing organic N to roots absorbing NO_3_^−^.

Our data thus show that absorbed organic N is preferentially used for root growth and that partitioning of biomass to roots is enhanced in the presence of organic N in the root medium. What remains to be explained is if, and how the two are connected, i.e. if and how the preferential allocation of absorbed organic N to root growth promotes an increase in root biomass. We have speculated that organic N would entail significant savings in terms of C for roots compared to that of inorganic N [31] and that this would enable a higher rate of root growth, all other things being equal. Such a mechanism would imply that the rate of root growth is at least partially a function of local soil conditions [2] and/or that the C:N status of roots is part of a signalling network that regulates biomass partitioning in plants [7].

Nitrogen availability exerts strong control of plant biomass partitioning and this response has been interpreted as plants maximising resource capture through allocating resources to the tissue in which the limiting resource is acquired [3]. More recent studies [42], [43] and reviews [7] have explored the mechanisms by which biomass partitioning is tuned by N availability. Using mutants with impaired capacity for NO_3_-reduction, Scheible et al. [44] showed that NO_3_^−^ concentrations of leaves exerted a strong impact on biomass partitioning, as well as on carbohydrate metabolism. However, the proposed role of NO_3_^−^ as a signal for shoot∶root partitioning has been challenged [43], partly on the basis that partitioning responses to shifts in N supply are similar for both NO_3_^−^ and NH_4_^+^. Experiments in which root systems are exposed to a spatially heterogeneous supply of N using split-root set-ups or with localised supply of N [36], [37], [45], show that root growth is stimulated in areas of high N. Zhang & Forde [46] described how localised supply of NO_3_^−^ stimulated initiation and growth of lateral roots. Thus, root growth is dependent on internal N status of plants but is also, to some degree, directly affected by the spatial distribution of soil N.

Different N forms exhibit highly variable diffusion coefficients (D_e_). Owen & Jones [24] estimated D_e_ for some different N forms in agricultural soils; values for NO_3_^−^, NH_4_^+^ and glycine were 0.3, 0.02 and 0.08 cm^2^ d^−1^, respectively. If N movement toward root surfaces is mainly through diffusion, these differences imply that plants need a smaller root surface area to acquire the same N uptake with NO_3_^−^ as a source compared with NH_4_^+^ and/or organic N [47]. Movement of N towards root surfaces may also occur via mass flow induced by transpiration. This mechanism would be especially important for NO_3_^−^ acquisition [48], [49], but less for less mobile ions, such as phosphate [50]. Thus, optimisation of the acquisition of mobile ions such as NO_3_^−^ could be functionally linked to preferential partitioning of biomass growth to above-ground tissues, while the opposite holds true for less mobile ions, including organic N compounds. There are also other potential links between NO_3_^−^ utilisation and shoot growth: reduction of NO_3_^−^ in the shoot is functionally linked to photosynthesis and may hence be assimilated and used for growth in above-ground tissues.

Under natural conditions, soil solution N concentrations co-vary with the chemical composition of soluble N. Hence; low N availabilities usually correspond to a large share of organic N in the soil solution while at increasing soil solution N concentrations, increasing shares of inorganic N and in particular NO_3_^−^ are found. Earlier studies have shown that plants are capable of competing with microbes for organic N substrates also under field conditions. Although the extent to which organic N is a significant N source for plants is still a matter of controversy, it is clear that plants do access such N forms in the field when available. A potential role of organic N in promoting root growth of plants under field conditions cannot, therefore, be dismissed. From the data presented in this study, we may speculate that the high values of root mass fraction of plants growing on poor soils may, to some extent result from an abundance of organic N in such soils while the gradual increase in above-ground biomass of plants inhabiting N rich soils may to some extent be promoted by higher rates of NO_3_^−^ availabilities on these sites. Unravelling the dependence of plant biomass partitioning on the abundance of organic and inorganic N sources under natural conditions will be a challenge for future studies.
